# Padé approximants for inverse trigonometric functions and their applications

**DOI:** 10.1186/s13660-017-1310-6

**Published:** 2017-02-01

**Authors:** Shanhe Wu, Gabriel Bercu

**Affiliations:** 1 Department of Mathematics, Longyan University, Longyan, Fujian 364012 P.R. China; 2 Department of Mathematics and Computer Sciences, ‘Dunărea de Jos’ University of Galaţi, 111 Domnească Street, Galaţi, 800201 Romania

**Keywords:** 41A21, 26D05, 26D15, 33B10, Padé approximant, inverse trigonometric functions, inequalities, refinement

## Abstract

The Padé approximation is a useful method for creating new inequalities and improving certain inequalities. In this paper we use the Padé approximant to give the refinements of some remarkable inequalities involving inverse trigonometric functions, it is shown that the new inequalities presented in this paper are more refined than that obtained in earlier papers.

## Introduction

The inequality

1.1 $$ \biggl(\frac{\sin x}{x} \biggr)^{2}+\frac{\tan x}{x}>2, \quad x \in \biggl(0, \frac{\pi}{2} \biggr), $$

referred to as the Wilker inequality, is one of the most famous inequalities for trigonometric functions.

The inverse trigonometric version of the Wilker inequality was considered in [1] and [2], as follows:

1.2 $$ \biggl(\frac{\arcsin x}{x} \biggr)^{2}+\frac{\arctan x}{x}>2, \quad x \in (0,1 ). $$

The Shafer-Fink double inequality for the arctangent function asserts that

1.3 $$ \frac{3x}{1+2\sqrt{1+x^{2}}}< \arctan x< \frac{\pi x}{1+2\sqrt{1+x^{2}}} $$

holds for any positive real number *x*.

Shafer’s inequality was recently generalized by Qi *et al.* in [3], as follows:

1.4 $$ \frac{ ( 1+a ) x}{a+\sqrt{1+x^{2}}}< \arctan x< \frac{ ( \frac{\pi}{2} ) x}{a+\sqrt{1+x^{2}}}, $$

where $0\leq a\leq\frac{1}{2}$ and $x>0$.

The Shafer-Fink double inequality for the arcsine function states that

1.5 $$ \frac{3x}{2+\sqrt{1-x^{2}}}\leq\arcsin x\leq\frac{\pi x}{2+\sqrt{1-x^{2}}} $$

holds for $0\leq x\leq1$. Furthermore, 3 and *π* are the best constants in (1.5).

For more relevant papers on the above topic, we refer the reader to [4–16] and the references therein.

It should be noted that these inequalities were proved using the variation of some functions and their derivatives, meanwhile, some of the above inequalities were improved using Taylor’s expansions of inverse trigonometric functions.

The Padé approximant is of the form of one polynomial divided by another polynomial, the technique was developed around 1890 by Henri Padé. It is well known that a Padé approximant is the ‘best’ approximation of a function by a rational function of given order. The rational approximation is particularly good for series with alternating terms and poor polynomial convergence (see [17–19]).

It is the aim of this paper to give the refinements of the aforesaid inverse trigonometric inequalities using the Padé approximant method.

Suppose that we are given a power series $\sum_{k=0}^{\infty}c_{k}x^{k}$ for representing a function $f ( x ) $, *i.e.*,

$$f(x)=c_{0}+c_{1}x+c_{2}x^{2}+ \cdots+c_{n}x^{n}+\cdots. $$

For a given function $f(x)$, the Padé approximant of order $[m/n]$ is the rational function

$$ R ( x ) =\frac{a_{0}+a_{1}x+\cdots+a_{m}x^{m}}{1+b_{1}x+\cdots +b_{n}x^{n}}, $$

which agrees with $f ( x ) $ at 0 to the highest possible order, *i.e.*,

$$ R ( 0 ) =f ( 0 ) ,\qquad R^{\prime} ( 0 ) =f^{\prime} ( 0 ),\qquad \ldots,\qquad R^{ ( m+n ) } ( 0 ) =f^{ ( m+n ) } ( 0 ) . $$

One can prove that the Padé approximant of a function $f ( x ) =\sum_{k=0}^{\infty}c_{k}x^{k}$ is unique for given *m* and *n*, that is, the coefficients $a_{0},a_{1},\ldots ,a_{m}$, $b_{1},b_{2},\ldots, b_{n}$ can be uniquely determined.

In fact, with the help of the Taylor series,

$$f(x)=\sum_{k=0}^{\infty}c_{k}x^{k}, \quad c_{k}=\frac{f^{(k)}(0)}{k!}, $$

the Padé approximant of $f(x)$ can be derived from the following relationship:

$$a_{0}+a_{1}x+\cdots+a_{m}x^{m}= \bigl(1+b_{1}x+\cdots +b_{n}x^{n}\bigr) \bigl(c_{0}+c_{1}x+\cdots+c_{m+n}x^{m+n} \bigr)+K(x)x^{m+n+1}, $$

where $K(x)$ is a polynomial factor.

For example, we consider the Taylor series

$$\arcsin x=x+\frac{x^{3}}{6}+O\bigl(x^{5}\bigr),\quad |x|< 1, $$

and its associate polynomial $x+{x^{3}}/{6} $.

Then the Padé approximant of arcsin*x* for the order $[1/2]$ is written as

$$\arcsin_{[1/2]}(x)=\frac{a_{0}+a_{1}x}{1+b_{1}x+b_{2}x^{2}}, $$

which satisfies

$$a_{0}+a_{1}x=\bigl(1+b_{1}x+b_{2}x^{2} \bigr) \biggl(x+\frac{x^{3}}{6}\biggr)+K(x)x^{4}. $$

From the above equation, we find

$$a_{0}=0, \qquad a_{1}=1, \qquad b_{1}=0,\qquad b_{2}=-\frac{1}{6}. $$

Therefore

$$\arcsin_{[1/2]}(x)=\frac{x}{1-\frac{1}{6}x^{2}}=\frac{6x}{6-x^{2}},\quad |x|< 1. $$

In Table 1 we provide a list of Padé approximants for arcsin*x* and arctan*x* which will be used in subsequent sections.

**Table 1:** **Padé approximants for** **arcsin** ***x*** **and** **arctan** ***x***

**Function**	**Padé approximant**	**Associate Taylor polynomials**
arcsin*x*	$\arcsin _{[1/2]}(x)=\frac{6}{6-x^{2}}$	$x+\frac{x^{3}}{6}$
arcsin*x*	$\arcsin_{[5/2]}(x)=\frac{61x^{5}+1\text{,}080x^{3}-2\text{,}520x}{1\text{,}500x^{2}-2\text{,}520}$	$x+\frac{x^{3}}{6}+\frac{3x^{5}}{40}+\frac{5x^{7}}{112} $
arctan*x*	$\arctan_{[1/2]}(x)=\frac{3x}{x^{2}+3}$	$x-\frac{x^{3}}{3} $
arctan*x*	$\arctan_{[3/2]}(x)=\frac{4x^{3}+15x}{9x^{2}+15}$	$x-\frac{x^{3}}{3}+\frac{x^{5}}{5}$
arctan*x*	$\arctan _{[5/2]}(x)=\frac{-4x^{5}+40x^{3}+105x}{75x^{2}+105}$	$x-\frac{x^{3}}{3}+\frac{x^{5}}{5}-\frac{x^{7}}{7}$
arctan*x*	$\arctan _{[3/4]}(x)=\frac{55x^{3}+105x}{9x^{4}+90x^{2}+105}$	$x-\frac{x^{3}}{3}+\frac{x^{5}}{5}-\frac{x^{7}}{7}$

## Some lemmas

In order to prove the main results in Section 3, we need the following lemmas.

### Lemma 2.1

*For every* $x\in ( 0,1 ) $, *one has*

2.1 $$ \arcsin x>\frac{61x^{5}+1\text{,}080x^{3}-2\text{,}520x}{1\text{,}500x^{2}-2\text{,}520}. $$

### Proof

We consider the function

$$f ( x ) =\arcsin x-\frac{61x^{5}+1\text{,}080x^{3}-2\text{,}520x}{1\text{,}500x^{2}-2\text{,}520}. $$

Differentiating $f(x)$ with respect to *x* yields

$$\begin{aligned} f^{\prime} ( x ) =&\frac{1}{\sqrt{1-x^{2}}}-\frac{305x^{6}+946x^{4}-4\text{,}872x^{2}+7\text{,}056}{4 ( 42-25x^{2} ) ^{2}} \\ =&\frac{4 ( 42-25x^{2} ) ^{2}-\sqrt{1-x^{2}} ( 305x^{6}+946x^{4}-4\text{,}872x^{2}+7\text{,}056 ) }{4\sqrt{1-x^{2}} ( 42-25x^{2} ) ^{2}} \\ =&\frac{16 ( 42-25x^{2} ) ^{4}- ( 1-x^{2} ) ( 305x^{6}+946x^{4}-4\text{,}872x^{2}+7\text{,}056 ) ^{2}}{4\sqrt{1-x^{2}} ( 42-25x^{2} ) ^{2} [ 4 ( 42-25x^{2} ) ^{2}+ ( 305x^{6}+946x^{4}-4\text{,}872x^{2}+7\text{,}056 ) \sqrt{1-x^{2}} ] } \\ =&\frac{x^{8} ( 93\text{,}025x^{6}+484\text{,}035x^{4}-2\text{,}654\text{,}064x^{2}+3\text{,}413\text{,}340 ) }{4\sqrt{1-x^{2}} ( 42-25x^{2} ) ^{2} [ 4 ( 42-25x^{2} ) ^{2}+ ( 305x^{6}+946x^{4}-4\text{,}872x^{2}+7\text{,}056 ) \sqrt{1-x^{2}} ] }. \end{aligned}$$

Evidently, $f^{\prime}(x)>0$ for $x\in ( 0,1 ) $. Then $f(x)$ is strictly increasing on $( 0,1 ) $. As $f ( 0 ) =0$, we get $f(x)>0$ for $x\in ( 0,1 ) $, this proves the validity of inequality (2.1). The proof of Lemma 2.1 is complete. □

### Lemma 2.2

*For every* $x\in ( 0,1 ) $, *one has*

2.2 $$ \arctan x>\frac{-4x^{5}+40x^{3}+105x}{75x^{2}+105}. $$

### Proof

We introduce a function $g: ( 0,1 ) \rightarrow \mathbb{R}$,

$$g ( x ) =\arctan x-\frac{-4x^{5}+40x^{3}+105x}{75x^{2}+105}. $$

Its derivative

$$\begin{aligned} g^{\prime} ( x ) =&\frac{1}{1+x^{2}}-\frac{(-4x^{6}+4x^{4}+21x^{2}+49)}{ ( 5x^{2}+7 ) ^{2}} \\ =&\frac{4x^{8}}{ ( 1+x^{2} ) ( 5x^{2}+7 ) ^{2}} \end{aligned}$$

is positive for every $x\in ( 0,1 ) $, therefore $g(x)$ is strictly increasing on $( 0,1 ) $. As $g ( 0 ) =0$, we have $g(x)>0$ for $( 0,1 ) $, which implies the desired inequality (2.2). Lemma 2.2 is proved. □

### Lemma 2.3

*For any positive real number* *x*, *one has*

2.3 $$ \frac{105x+55x^{3}}{105+90x^{2}+9x^{4}}< \arctan x< \frac{15x+4x^{3}}{15+9x^{2}}. $$

### Proof

We define a function $\psi: ( 0,\infty ) \rightarrow \mathbb{R}$ by

$$\psi ( x ) =\arctan x-\frac{15x+4x^{3}}{15+9x^{2}}. $$

Then we have

$$\psi^{\prime} ( x ) =\frac{-4x^{6}}{ ( 1+x^{2} ) ( 5+3x^{2} ) ^{2}}< 0 $$

for $x \in ( 0,\infty ) $, thus *ψ* is strictly decreasing on $( 0,\infty ) $. It follows from $\psi(0) =0$ that $\psi(x)<0$ for $x\in (0,\infty ) $.

Let

$$\phi ( x ) =\arctan x-\frac{105x+55x^{3}}{105+90x^{2}+9x^{4}},\quad x\in ( 0,\infty ). $$

Differentiating $\phi(x)$ with respect to *x* gives

$$ \phi^{\prime} ( x ) =\frac{64x^{8}}{ ( 1+x^{2} ) ( 35+30x^{2}+3x^{4} ) ^{2}}. $$

We have $\phi^{\prime}(x)>0$ for $( 0,\infty ) $, hence *ϕ* is strictly increasing on $( 0,\infty ) $. Since $\phi ( 0 ) =0$, we deduce that $\phi(x)>0$ for $x \in ( 0,\infty ) $.

The inequality (2.3) is proved. This completes the proof of Lemma 2.3. □

## Main results

In this section we will formulate and prove the refinements of the aforesaid inverse trigonometric inequalities.

### Theorem 3.1

*For every* $0<\vert x\vert <1$, *the following inequality holds*:

3.1 $$ \biggl( \frac{\arcsin x}{x} \biggr) ^{2}+ \frac{\arctan x}{x}>\frac{A ( x ) }{3\text{,}600 ( 5x^{2}+7 ) ( 25x^{2}-42 ) ^{2}}>2, $$

*where* $A ( x ) =18\text{,}605x^{10}+84\text{,}847x^{8}+13\text{,}233\text{,}120x^{6}-27\text{,}306\text{,}720x^{4}-42\text{,}336\text{,}000x^{2}+88\text{,}905\text{,}600$.

### Proof

We remark that if the above inequality is true for $x\in ( 0,1 ) $, then it holds clearly for $x\in ( -1,0 ) $. Therefore, it is sufficient to prove only for $x\in ( 0,1 ) $.

By using Lemmas 2.1 and 2.2, we have

$$\begin{aligned} \biggl( \frac{\arcsin x}{x} \biggr) ^{2}+\frac{\arctan x}{x} >& \biggl( \frac{61x^{4}+1\text{,}080x^{2}-2\text{,}520}{1\text{,}500x^{2}-2\text{,}520} \biggr) ^{2}+\frac {-4x^{4}+40x^{2}+105}{75x^{2}+105} \\ =&\frac{A ( x ) }{3\text{,}600 ( 5x^{2}+7 ) ( 25x^{2}-42 ) ^{2}}. \end{aligned}$$

Additionally,

$$\begin{aligned}& \frac{A ( x ) }{3\text{,}600 ( 5x^{2}+7 ) ( 25x^{2}-42 ) ^{2}}-2 \\& \quad =\frac{x^{4} ( 18\text{,}605x^{6}+84\text{,}847x^{4} -9\text{,}266\text{,}880x^{2}+16\text{,}793\text{,}280 )}{3\text{,}600 ( 5x^{2}+7 ) ( 25x^{2}-42 )^{2}}>0, \end{aligned}$$

which implies the desired inequality (3.1). The proof of Theorem (3.1) is completed. □

### Remark 1

By making use of the Taylor expansion, we have

3.2 $$ \biggl( \frac{\arcsin x}{x} \biggr) ^{2}+ \frac{\arctan x}{x}=2+\frac{17}{45}x^{4}-\frac{1}{35}x^{6}+O \bigl(x^{8}\bigr). $$

We note that

$$\begin{aligned}& \frac{A ( x ) }{3\text{,}600 ( 5x^{2}+7 ) ( 25x^{2}-42 ) ^{2}}- \biggl( 2+\frac{17}{45}x^{4}- \frac{1}{35}x^{6} \biggr) \\& \quad = \frac{x^{8} ( 250\text{,}000x^{4}-3\text{,}781\text{,}085x^{2}+6\text{,}074\text{,}481 ) }{2\text{,}800 ( 42-25x^{2} ) ^{2} ( 5x^{2}+7 ) }>0 \end{aligned}$$

for $0<\vert x\vert <1$. Hence the Padé approximation provides a better inequality than the inequality below

3.3 $$ \biggl( \frac{\arcsin x}{x} \biggr) ^{2}+ \frac{\arctan x}{x}> 2+\frac{17}{45}x^{4}-\frac{1}{35}x^{6}. $$

The Taylor’s approximation prompts to consider another method for proving the inequality asserted in Theorem 3.1, that is, we may add more terms to the Taylor polynomial (3.2) in order to prove the inequality (3.1). However, in this way, we will face complicated calculations on the high-order derivatives of $((\arcsin x)/{x})^{2}+{(\arctan x)}/{x}$ and some cumbersome inequalities resulting from it.

### Theorem 3.2

*Let* $0\leq a\leq\frac{1}{2}$. *Then for* $0< x<1$ *we have the following inequality*:

3.4 $$ \frac{ ( 1+a ) x}{a+\sqrt{1+x^{2}}}< \frac{105x+55x^{3}}{105+90x^{2}+9x^{4}}< \arctan x< \frac{15x+4x^{3}}{15+9x^{2}}< \frac{ ( \frac{\pi }{2} ) x}{a+\sqrt{1+x^{2}}}. $$

### Proof

The first inequality can be rewritten as

$$ 105+ ( 90+35a ) x^{2}+9 ( 1+a ) x^{4}< \sqrt {1+x^{2}} \bigl( 105+55x^{2} \bigr) . $$

Since $0\leq a\leq\frac{1}{2}$, it is easy to find that

$$105+ ( 90+35a ) x^{2}+9 ( 1+a ) x^{4}\leq \frac {210+215x^{2}+27x^{4}}{2}. $$

Thus we need to prove the following inequality:

$$ \frac{210+215x^{2}+27x^{4}}{2}< \sqrt{1+x^{2}} \bigl( 105+55x^{2} \bigr) $$

or, equivalently,

$$\bigl(210+215x^{2}+27x^{4}\bigr)^{2}< 4 \bigl(1+x^{2}\bigr) \bigl( 105+55x^{2} \bigr) ^{2}, $$

which can be deduced from a simple calculation:

$$\begin{aligned} & \bigl(210+215x^{2}+27x^{4}\bigr)^{2}-4 \bigl(1+x^{2}\bigr) \bigl( 105+55x^{2} \bigr) ^{2} \\ &\quad =x^{4} \bigl( 729x^{4}-490x^{2}-735 \bigr) \\ &\quad =x^{4} \bigl[ - \bigl( 729x^{2}+239 \bigr) ( 1-x ) (1+x)-496 \bigr] \\ &\quad < 0. \end{aligned}$$

Therefore, we obtain

3.5 $$ \frac{ ( 1+a ) x}{a+\sqrt{1+x^{2}}}< \frac{105x+55x^{3}}{105+90x^{2}+9x^{4}}. $$

The last inequality can be rewritten as

$$ \bigl( 15+4x^{2} \bigr) \sqrt{1+x^{2}}< \biggl( \frac{15\pi}{2}-15a \biggr) + \biggl( \frac{9\pi}{2}-4a \biggr) x^{2}. $$

Since $0\leq a\leq\frac{1}{2}$, it is easy to observe that

$$\biggl(\frac{15\pi}{2}-15a \biggr) + \biggl( \frac{9\pi}{2}-4a \biggr) x^{2}\geq\frac{15\pi-15}{2}+\frac{9\pi-4}{2}x^{2}. $$

Hence we need to prove the following inequality:

$$\frac{15\pi-15}{2}+\frac{9\pi-4}{2}x^{2}> \bigl( 15+4x^{2} \bigr) \sqrt{1+x^{2}}$$

or, equivalently,

$$\bigl(15\pi-15+(9\pi-4)x^{2}\bigr)^{2}>4 \bigl( 15+4x^{2} \bigr) ^{2}\bigl(1+x^{2}\bigr). $$

Now, from

$$\begin{aligned} & \bigl(15\pi-15+(9\pi-4)x^{2}\bigr)^{2}-4 \bigl( 15+4x^{2} \bigr) ^{2}\bigl(1+x^{2}\bigr) \\ &\quad = \bigl( 1-x^{2} \bigr) \bigl[64x^{4}+ \bigl( 72 \pi-81\pi ^{2}+592 \bigr) \bigl(1+x^{2}\bigr) \bigr] \\ &\qquad {} + \bigl( 270\pi^{2}-390\pi-1\text{,}260 \bigr) x^{2}+ \bigl( 306\pi^{2}-522\pi-1267 \bigr), \end{aligned}$$

together with

$$72\pi-81\pi^{2}+592>0, \qquad 270\pi^{2}-390\pi-1 \text{,}260>0,\qquad 306\pi^{2}-522\pi-1\text{,}267>0, $$

we conclude that

$$\bigl(15\pi-15+(9\pi-4)x^{2}\bigr)^{2}-4 \bigl( 15+4x^{2} \bigr) ^{2}\bigl(1+x^{2}\bigr)>0, $$

which leads to the inequality

3.6 $$ \frac{15x+4x^{3}}{15+9x^{2}}< \frac{ ( \frac{\pi}{2} ) x}{a+\sqrt{1+x^{2}}}. $$

In conclusion, the inequality (3.4) follows immediately from inequalities (3.5), (3.6), and the inequality (2.3) given by Lemma 2.3. This completes the proof of Theorem 3.2. □

Finally, we deal with the improved version of the Shafer-Fink inequality (1.5).

Using the well-known trigonometric identity

$$ 2\arctan x=\arcsin\frac{2x}{1+x^{2}},\quad x\in ( 0,1 ), $$

and the double inequality from the Lemma 2.3, we obtain

$$\frac{210x+110x^{3}}{105+90x^{2}+9x^{4}}< \arcsin\frac {2x}{1+x^{2}}< \frac{30x+8x^{3}}{15+9x^{2}}. $$

Substituting the expression $\frac{2x}{1+x^{2}}$ by *y* ($0< y<1$), we get

$$\begin{aligned}& \frac{210 (\frac{1-\sqrt{1-y^{2}}}{y} )+110 ( \frac{1-\sqrt {1-y^{2}}}{y} ) ^{3}}{105+90 ( \frac{1-\sqrt{1-y^{2}}}{y} )^{2} +9 ( \frac{1-\sqrt{1-y^{2}}}{y} ) ^{4}} \\& \quad < \arcsin y< \frac{30 (\frac{1-\sqrt{1-y^{2}}}{y} )+8 ( \frac{1-\sqrt{1-y^{2}}}{y} ) ^{3}}{15+9 ( \frac{1-\sqrt{1-y^{2}}}{y} ) ^{2}}. \end{aligned}$$

After some elementary calculations, the above inequality can be transformed to the following refinement of Shafer’s inequality for the arcsine function.

### Theorem 3.3

*For every* $x\in ( 0,1 ) $, *one has*

3.7 $$ \frac{x ( 80+25\sqrt{1-x^{2}} ) }{57-6x^{2}+48\sqrt{1-x^{2}}}< \arcsin x< \frac{x ( 19+11\sqrt{1-x^{2}} ) }{3 ( 1+\sqrt{1-x^{2}} ) ( 4+\sqrt{1-x^{2}} ) } . $$

### Proof

For the sake of completeness, besides the solution above, we will provide another elementary solution in regard to the inequalities (3.7).

Putting $\arcsin x=t$, $t\in ( 0,\frac{\pi}{2} ) $ in (3.7) gives

3.8 $$ \frac{(\sin t) ( 80+25\cos t ) }{57-6\sin^{2}t+48\cos t}< t< \frac{(\sin t) ( 19+11\cos t ) }{3 ( 1+\cos t ) ( 4+\cos t ) }. $$

The left-hand side inequality of (3.8) can be rewritten as

$$ 108t+96t\cos t+6t\cos2t-160\sin t-25\sin2t>0. $$

Let us consider the function $u: ( 0,\frac{\pi}{2} ) \rightarrow\mathbb{R} $,

$$u ( t ) =108t+96t\cos t+6t\cos2t-160\sin t-25\sin2t. $$

Its derivatives are

$$\begin{aligned}& u^{\prime} ( t ) =108-64\cos t-96t\sin t-12t\sin2t-44\cos 2t, \\& u^{\prime\prime} ( t ) =-32\sin t+76\sin2t-96t\cos t-24t\cos2t, \end{aligned}$$

and

$$\begin{aligned} u^{ ( 3 ) } ( t ) =&128 ( -\cos t+\cos2t ) +96t\sin t+96t\sin t\cos t \\ =&64 \biggl(\sin\frac{t}{2} \biggr) \biggl( -4\sin \frac{3t}{2}+3t \cos\frac{t}{2}+3t\cos t\cos\frac{t}{2} \biggr) \\ =&64 \biggl(\sin\frac{t}{2} \biggr)v(t). \end{aligned}$$

Using the formula

$$ \sin3\alpha=3\sin\alpha-4\sin^{3}\alpha, $$

the function $v(t)$ can be further rearranged as

$$ v ( t ) =4\sin\frac{t}{2}-16\sin\frac{t}{2}\cos^{2} \frac {t}{2}+6t\cos^{3}\frac{t}{2}. $$

Then

$$\begin{aligned} v^{\prime} ( t ) =&2\cos\frac{t}{2}-2\cos^{3} \frac{t}{2}+16\cos\frac{t}{2}\sin^{2} \frac{t}{2}-9t\cos^{2}\frac{t}{2}\sin\frac {t}{2} \\ =&9 \biggl(\sin\frac{t}{2}\cos\frac{t}{2} \biggr) \biggl( 2\sin \frac {t}{2}-t\cos\frac{t}{2} \biggr) \\ =&9 \biggl(\sin\frac{t}{2}\cos\frac{t}{2} \biggr)w(t). \end{aligned}$$

Note that

$$w^{\prime} ( t ) =\frac{t}{2}\sin\frac{t}{2}>0,\quad t\in \biggl( 0,\frac{\pi}{2} \biggr). $$

Therefore $w(t)$ is strictly increasing on $( 0,\frac{\pi }{2} ) $. As $w ( 0 ) =0$, it follows that $w(t)>0$ for $t \in ( 0,\frac{\pi}{2} ) $. Hence $v^{\prime } ( t ) >0$ for $t\in ( 0,\frac{\pi}{2} ) $.

Using similar arguments, we have

$$v(t)>0,\qquad u^{ ( 3 ) }(t)>0,\qquad u^{\prime\prime}(t) >0,\qquad u^{\prime }(t)>0,\qquad u(t)>0 $$

for $t \in ( 0,\frac{\pi}{2} ) $. The left-hand side inequality of (3.8) is proved.

In order to prove the right-hand side inequality of (3.8), we observe that

$$ \frac{\sin t}{1+\cos t}=\frac{1-\cos t}{\sin t}. $$

It is easy to find that the right-hand side inequality of (3.8) is equivalent to the following inequality:

$$ 24t\sin t+3t\sin2t+16\cos t+11\cos2t-27< 0. $$

Define a function $s: ( 0,\frac{\pi}{2} ) \rightarrow \mathbb{R}$ by

$$s ( t ) =24t\sin t+3t\sin2t+16\cos t+11\cos2t-27. $$

Differentiating $s(t)$ with respect to *t* gives

$$\begin{aligned} \begin{aligned} &s^{\prime} ( t ) = 8\sin t+24t\cos t-19\sin2t+6t\cos 2t, \\ &s^{\prime\prime } ( t ) = 32 ( \cos t-\cos 2t ) -24t(\sin t) ( 1+\cos t ) \\ &\hphantom{s^{\prime\prime } ( t )}= 32 \biggl(\sin\frac{t}{2} \biggr) \biggl( 2\sin\frac {3t}{2}-3t \cos^{3}\frac{t}{2} \biggr) \\ &\hphantom{s^{\prime\prime } ( t )} = 32 \biggl(\sin\frac{t}{2} \biggr) \biggl( 6\sin\frac{t}{2}-8 \sin ^{3}t-3t\cos^{3}\frac{t}{2} \biggr) \\ &\hphantom{s^{\prime\prime } ( t )} = 32 \biggl(\sin\frac{t}{2} \biggr)r(t). \end{aligned} \end{aligned}$$

The function $r ( t ) $ has the derivative

$$\begin{aligned} r^{\prime} ( t ) =&9 \biggl(\sin\frac{t}{2}\cos\frac {t}{2} \biggr) \biggl( \frac{1}{2}t\cos\frac{t}{2}-\sin\frac{t}{2} \biggr) \\ =&9 \biggl(\sin\frac{t}{2}\cos\frac{t}{2} \biggr)p(t). \end{aligned}$$

Also, we have

$$p^{\prime} ( t ) =-\frac{t}{4}\sin\frac{t}{2}< 0, \quad t\in \biggl( 0,\frac{\pi}{2} \biggr). $$

Since $p ( 0 ) =0$, it follows that $p(t)<0$ for $t \in ( 0,\frac{\pi}{2} ) $. Therefore $r^{\prime}(t)<0$ for $t\in ( 0,\frac{\pi}{2} ) $.

Using the same arguments, we have

$$r(t)< 0,\qquad s^{\prime\prime}(t)< 0,\qquad s^{\prime}(t)< 0, \qquad s(t)< 0 $$

for $t \in ( 0,\frac{\pi}{2} )$, the right-hand side inequality of (3.8) is proved. The proof of Theorem 3.3 is completed. □

### Remark 2

It is easy to observe that

$$\begin{aligned} & \frac{3x}{2+\sqrt{1-x^{2}}}-\frac{x(80+25\sqrt{1-x^{2}})}{57-6x^{2}+48\sqrt{1-x^{2}}} \\ &\quad =\frac{ 7x ( 2\sqrt{1-x^{2}}+x^{2}-2 ) }{(2+\sqrt{1-x^{2}})(57-6x^{2}+48\sqrt{1-x^{2}})} \\ & \quad =\frac{-7x^{5}}{(2+\sqrt{1-x^{2}})(57-6x^{2}+48\sqrt{1-x^{2}}) ( 2\sqrt{1-x^{2}}+2-x^{2} ) } \\ &\quad < 0. \end{aligned}$$

Thus, the left-hand side inequality of (3.7) is stronger than the left-hand side inequality of (1.5).

Also, we have

$$\begin{aligned} & \frac{x(19+11\sqrt{1-x^{2}})}{3(1+\sqrt{1-x^{2}})(4+\sqrt{1-x^{2}})}-\frac{\pi x}{2+\sqrt{1-x^{2}}} \\ &\quad =\frac{x ( 3\pi x^{2}-11x^{2}+49-15\pi-\sqrt{1-x^{2}} ( 15\pi-41 ) ) }{3(1+\sqrt{1-x^{2}})(4+\sqrt{1-x^{2}})(2+\sqrt{1-x^{2}})} \end{aligned}$$

and

$$\begin{aligned} & \bigl(3\pi x^{2}-11x^{2}+49-15\pi\bigr)^{2}- \bigl(1-x^{2}\bigr) ( 15\pi-41 ) ^{2} \\ &\quad = \bigl( 9\pi^{2}-66\pi+121 \bigr) x^{4}+ \bigl( 135 \pi ^{2}-606\pi+603 \bigr) x^{2}+720-240\pi \\ &\quad =(x-C_{1}) (x+C_{1}) \bigl(x^{2}+C_{2} \bigr), \end{aligned}$$

where

$$\begin{aligned}& C_{1} =\sqrt{ \frac{606\pi-135\pi^{2}-603+ ( 15\pi-41 ) \sqrt{ 81\pi^{2}-246\pi+9}}{ 2 ( 3\pi-11 ) ^{2}}}\approx 0.99876, \\& C_{2} =\frac{606\pi-135\pi^{2}-603- ( 15\pi-41 ) \sqrt { 81\pi^{2}-246\pi+9}}{ 2 ( 3\pi-11 ) ^{2}}\approx13.729. \end{aligned}$$

Hence we conclude that

$$\frac{x(19+11\sqrt{1-x^{2}})}{3(1+\sqrt{1-x^{2}})(4+\sqrt{1-x^{2}})}\leq\frac{\pi x}{2+\sqrt{1-x^{2}}}$$

holds for $0< x<0.99876$, which implies that the right-hand side inequality of (3.7) is stronger than the right-hand side inequality of (1.5) when $0< x<0.99876$.

## Conclusions

The Padé approximation is a useful method for creating new inequalities and improving certain inequalities. Firstly, we introduce the main technique of Padé approximation and establish some Padé approximants for arcsin*x* and arctan*x*. And then we use the Padé approximation to improve some remarkable inequalities involving inverse trigonometric functions, we show that the new inequalities presented in this paper are more refined than that obtained in earlier papers. It is worth to mention that the Padé approximation method has also been applied to dealing with the refinements of certain inequalities for trigonometric functions and hyperbolic functions in our recent papers [20] and [21]. We expect that the method will be useful in solving some others problems concerning inequalities.
